# Influence of age and sex on taste function of healthy subjects

**DOI:** 10.1371/journal.pone.0227014

**Published:** 2020-06-12

**Authors:** Jing-Jie Wang, Kai-Li Liang, Wen-Jiun Lin, Chih-Yi Chen, Rong-San Jiang

**Affiliations:** 1 Institute of Medicine, Chung Shan Medical University, Taichung, Taiwan; 2 Department of Otolaryngology, Taichung Veterans General Hospital, Taichung, Taiwan; 3 Faculty of Medicine, National Yang-Ming Medical University, Taipei, Taiwan; 4 School of Medicine, Chung Shan Medical University, Taichung, Taiwan; 5 Division of Thoracic Surgery, Chung Shan Medical University Hospital, Taichung, Taiwan; 6 Department of Medical Research, Taichung Veterans General Hospital, Taichung, Taiwan; 7 Rong Hsing Research Center for Translational Medicine, National Chung Hsing University, Taichung, Taiwan; Tohoku University, JAPAN

## Abstract

The purpose of this study was to determine the influence of age and sex on the taste functions of healthy Taiwanese. Subjects were divided into groups based on their sex and age: 20–39 years, 40–59 years, or ≥ 60 years. We evaluated the taste functions of subjects using the whole mouth suprathreshold taste test and the taste quad test. For the whole-mouth test, subjects were instructed to sip and swish sweet, sour, salty, and bitter solutions, each at 5 different suprathreshold concentrations. Each subject was required to indicate the taste quality, and to rate the intensity and unpleasantness/pleasantness of each taste of the solutions. For the quad test, the 4 quadrants of the tongue surface were tested by applying a drop of one concentration of sweet, sour, salty, or bitter solutions 6 times. Subjects then indicated the taste quality and rated the intensity of the solution. We found that in the whole mouth test, the total correct identification score dropped with age, but the ability to identify sweet and salty qualities was not affected by age. No differences were found between males and females, except women scored better than men for sweetness in the 40–59 years age group. The intensity rating scores were higher in the 20–39 years age group, regardless of sex. With regard to the pleasantness of tastants, female subjects in the 20–39 years age group found sweet solution more pleasant than the older subjects did. In the quad test, the total correct identification score decreased with age, but there were no differences between males and females. Thus, our findings showed that both age and sex affected the taste functions of healthy Taiwanese to some extent, and differences were dependent on tongue region and taste quality.

## Introduction

Taste dysfunction is estimated to affect 26.3 million people in the US according to a 2016 nationwide survey [1]. It is generally accepted that smell ability in humans declines with age [2]. However, the effect of aging on taste function is considered small and varies among individuals [3]. A systemic review of the effects of aging on taste function reported that taste perception declines with age, although the extent of decline differs across studies [4]. It is also known that taste preference, detection threshold, and reactivity to taste stimuli might differ between males and females [5]. However, the exact nature of these sex differences remains undetermined [5]. Gudziol and Hummel [6] used the ‘three-drop test’ to study taste function in a population of Europeans and found that taste functions in women were more sensitive than in men. Another study also reported that sex affects the perception of sour and bitter tastes [7].

Taste function has rarely been investigated in Asian populations. Yong et al. [8] investigated the effect of age and sex on taste function in 90 healthy Chinese adults, using the same method of Gudziol and Hummel [6]. They found no effects of age or sex on taste function. They proposed that eating habits may influence taste results, and that Asians are more sensitive to tastants. However, in their study, only subjects <65 years were analyzed.

Taste function has been determined using both chemical and electrical stimuli. Several methods have been developed to present chemical stimuli to human subjects, including ‘sipping & spitting’, tastant strips, taste tablets, cotton swabs, and discs [9–11]. Solution-based taste tests have known reliability [6]. In general, there are two types of taste tests: the whole mouth test and the regional test [12]. The whole mouth test provides general taste sensitivity [13], while the regional test can detect gustatory blind regions on the tongue [14]. In order to further clarify the influence of age and sex on taste functions, specifically in an Asian population, we investigated taste function in healthy Taiwanese, using the solution-based taste tests.

## Materials and methods

### Ethical statements

This study was approved by the Institutional Review Board of Taichung Veterans General Hospital, Taiwan (IRB number: CF18048A). Informed written consent was obtained from all enrolled subjects.

### Study subjects

Healthy Taiwanese volunteers with a normal self-rated taste function were enrolled. We excluded subjects with a history of oral or middle ear surgery, or acute oral infections. Study subjects were divided into three age groups: 20–39 years, 40–59 years, and ≥60 years. The grouping criteria were based on a previous study conducted by Gudziol and Hummel [6]. Forty male and 40 females were enrolled for each age group. A total of 240 subjects participated in this study. They each performed the whole mouth suprathreshold taste test and the taste quad test to measure their taste function.

### Taste tests

Two solution-based taste tests, the whole mouth suprathreshold test, and the taste quad test, were used for evaluation of taste function following the methods used at the Smell & Taste Center of the University of Pennsylvania [15]. Between these two tests, subjects were allowed a break of 10 minutes.

#### Whole mouth suprathreshold taste test

Five different suprathreshold concentrations of 4 basic tastant solutions were used in the whole mouth suprathreshold taste test. The concentrations of tastant solutions followed the methods of Doty et al. [15]. Powders of sucrose, citric acid, sodium chloride (I Chan chemical Ltd., Taipei, Taiwan), and caffeine (Uni-Onward Corp., New Taipei City, Taiwan) were individually dissolved in distilled water to prepare the following tastant solutions: sweet solution (concentrations of sucrose, S1-S5: 0.08, 0.16, 0.32, 0.64, 1.28 mol/L), sour solution (concentrations of citric acid, A1-A5: 0.0026, 0.0051, 0.0102, 0.0205, 0.0410 mol/L), salty solution (concentrations of sodium chloride, N1-N5: 0.032, 0.064, 0.128, 0.256, 0.512 mol/L), and bitter solution (concentrations of caffeine, C1-C5: 0.0026, 0.0051, 0.0102, 0.0205, 0.0410 mol/L).

**Fig 1:**
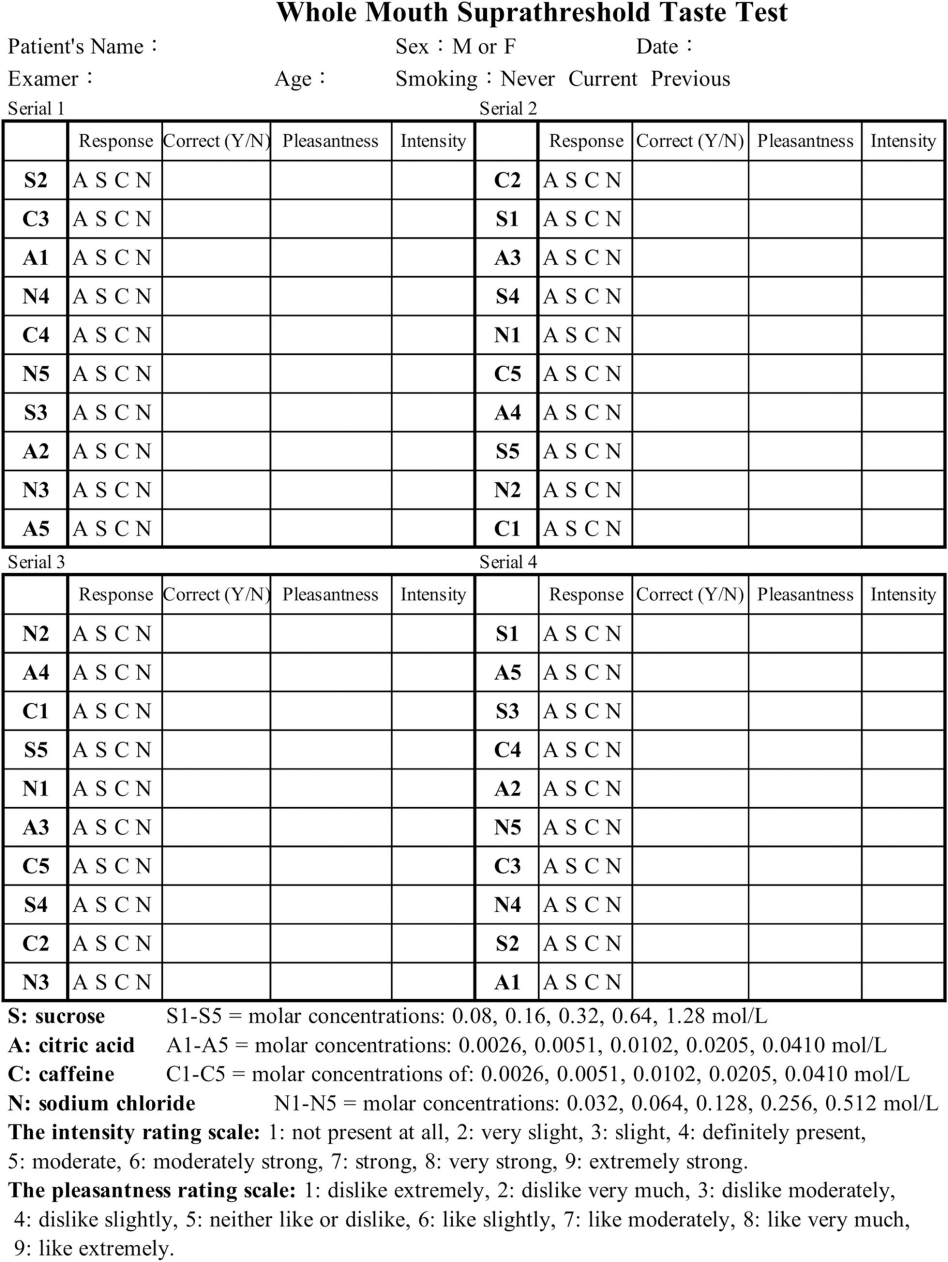
Recording data sheet of the whole mouth suprathreshold taste test.

Prior to the beginning of the test, each subject was instructed not to smoke or eat for at least one hour. A small cup containing 10 mL of each tastant solution was then presented to the subject in a counterbalanced order. The presentation order is shown in Fig 1 (tastant solutions presented from serial 1 to serial 4). The solution in the cup was sipped, swished in the mouth for 10 seconds, and expectorated. The subject was then asked to select one of the 4 tastes (sweet, sour, bitter, or salty) for each solution, and to make a best guess if in doubt. One point was scored if a correct identification of the taste was made. The subject was also instructed to rate the intensity and unpleasantness/pleasantness of each solution, as follows. The intensity of the solution was rated using a 9-point scale: 1: not present at all, 2: very slight, 3: slight, 4: definitely present, 5: moderate, 6: moderately strong; 7: strong; 8: very strong; 9: extremely strong. The pleasantness of the solution was also rated using a 9-point scale: 1: dislike extremely, 2: dislike very much, 3: dislike moderately, 4: dislike slightly, 5: neither like nor dislike, 6: like slightly; 7: like moderately; 8: like very much, 9: like extremely. Between successive cups, the subject was instructed to rinse his or her mouth with distilled water. Each of the 5 suprathreshold concentrations for the 4 tastant solutions was tested twice by the subject. Therefore, a total of 40 tests (4 tastants × 5 concentrations × 2 trials) were performed to generate a maximum correct quality identification score of 40. The recording data sheet of the whole mouth test is shown in Fig 1.

#### Taste quad test

**Fig 2:**
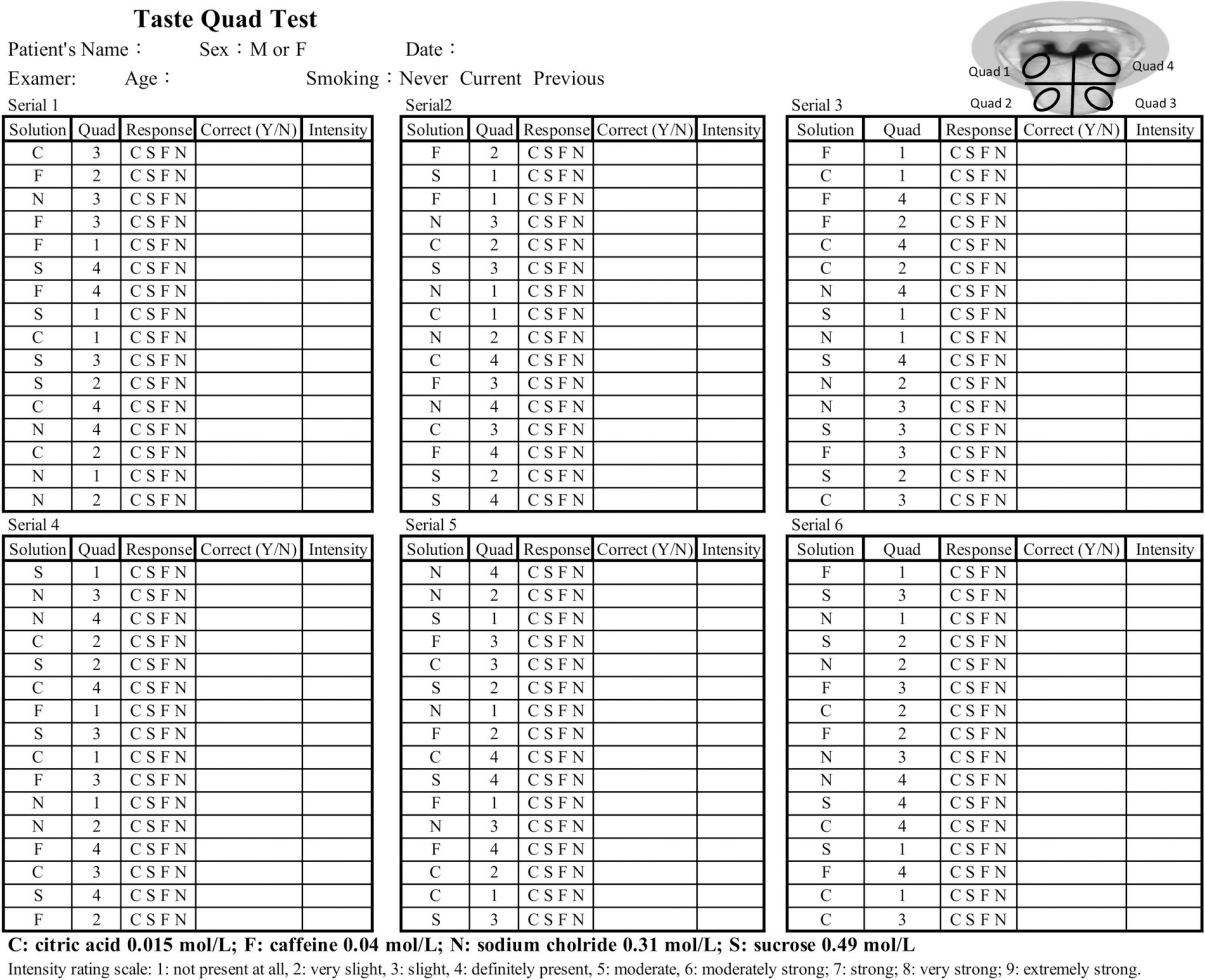
Recording data sheet of taste quad test.

In this study, a single suprathreshold concentration of solution was prepared for each of the 4 basic tastants as follows: a 0.49 mol/L sucrose solution (sweet, **S**), a 0.015 mol/L citric acid solution (sour, **C**), a 0.31 mol/L sodium chloride solution (salty, **N**), and a 0.04 mol/L caffeine solution (bitter, **F**). Prior to beginning the test, the subject was instructed to protrude his or her tongue, which was visually divided by the tester into 4 quadrants (quadrant 1: right posterior tongue, quadrant 2: right anterior tongue, quadrant 3: left anterior tongue, and quadrant 4: left posterior tongue, Fig 2). Next, using a micropipette, the tester applied a drop (15 μL) of the tastant solution onto one of the 4 quadrants. The subject indicated which taste (sweet, sour, bitter, or salty) the solution was presented, and to make a best guess if in doubt. The subject also rated the intensity of the solution using the same 9-point scale that was used in the whole mouth test. Then, the subject rinsed with distilled water. On each tongue quadrant, 4 tastant solutions were tested 6 times in a counterbalanced order (tastant solutions presented from serial 1 to serial 6). One point was scored if a correct identification of the taste was made. Therefore, a total of 96 tests were performed for each subject to generate a maximum score of 96. The recording data sheet of the quad test is shown in Fig 2.

### Statistical analyses

Descriptive data were presented as median, minimum, maximum, and percentiles. The Kolmogorov-Smirnov test was used to test for data normality. In both the whole mouth test and quad test, the correct quality identification scores, intensity, and unpleasantness/pleasantness rating scores were compared across age groups using the Kruskal-Wallis test, and then compared between two groups using the Dunn-Bonferroni test. The correct quality identification scores of the tastant solutions in each age group were compared across the 4 tongue quadrants using the Friedman test. All computations were performed using SPSS version 20 (Armonk, NY: IBM Corp.). Two-tailed p-values <0.05 were considered to be statistically significant.

## Results

A total of 240 study subjects were enrolled. Subjects were divided into 6 groups: males aged 20–30 (median: 25.5) years, males aged 40–59 (median: 49.5) years, males aged ≥60 (median: 66.5) years, females aged 20–30 (median: 28) years, females aged 40–59 (median: 50) years, and females aged ≥60 (median: 64) years. There were 40 subjects in each group.

### Whole mouth suprathreshold taste test

**Fig 3:**
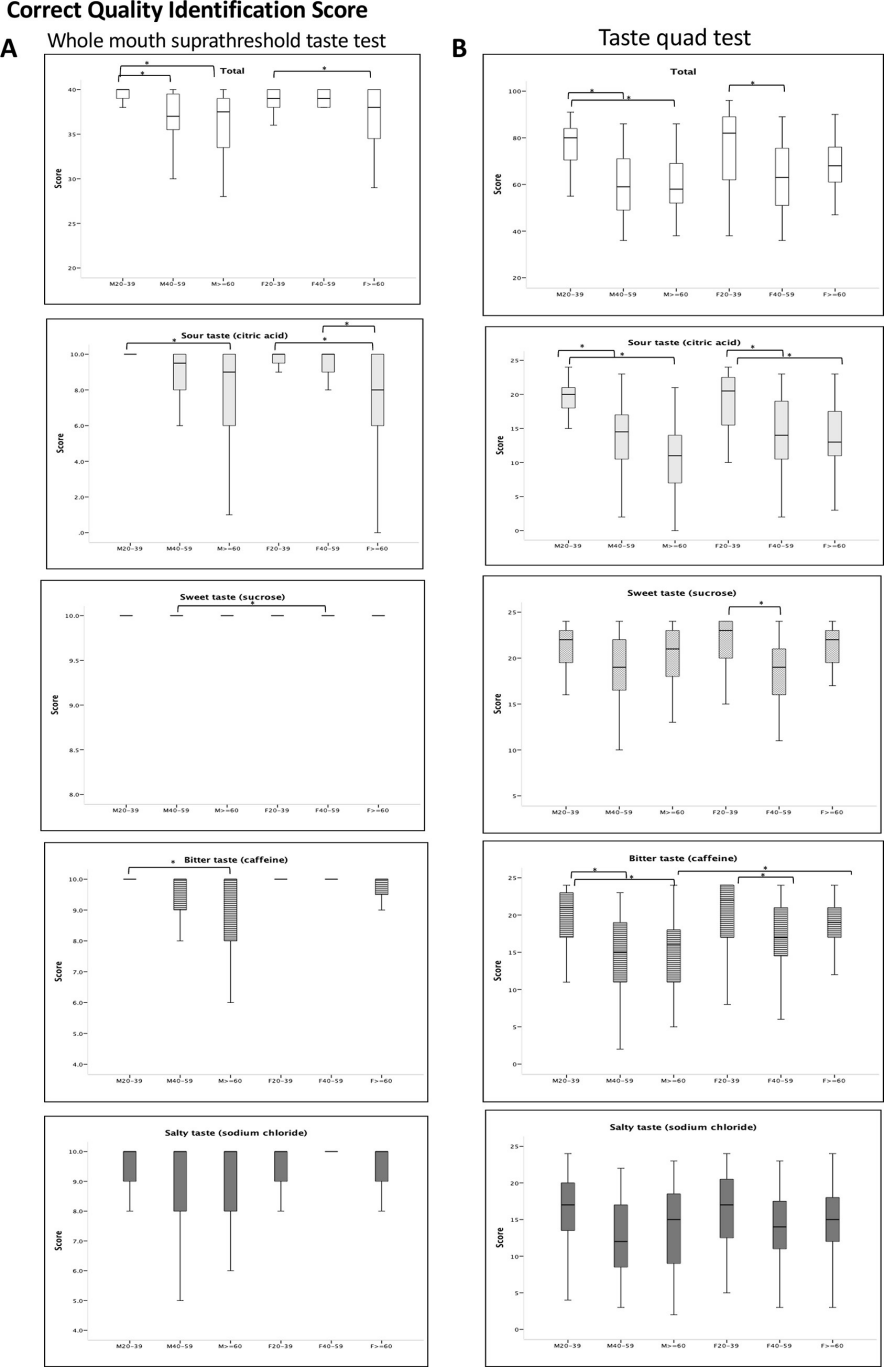
The influence of age and sex on taste function. (A) the whole mouth suprathreshold test; (B) the taste quad test. In the plots, the boxes depict the 25th, 50th, and 75th quartiles. The lines extending parallel from the boxes are used to indicate the range of correct quality identification score. *Significant differences (p<0.05) between two groups.

The correct quality identification scores of all and each group are shown in Table 1. The total scores of correct quality identification of the 4 tastant solutions were significantly higher for the 20–39 years age group than for the other age groups (20–39 years vs. 40–59 years: p = 0.01 for males; 20–39 years vs. ≥60 years: p<0.001 for males, p = 0.033 for females). For individual tastants, male subjects in the 20–39 years age group had significantly higher scores for sour and bitter solutions than males aged ≥60 years (p<0.001 for sour; p = 0.004 for bitter). Female subjects in the ≥60 years age group had significantly lower scores for the sour solution than those of younger subjects (vs. 20–39 years, p = 0.001; vs. 40–59 years, p = 0.02). No differences in correct quality identification scores were found between male and female subjects of the same age group for sour, bitter, and salty tastes, but for sweetness, females had higher scores than males in the 40–59 years age group (p = 0.047) (Fig 3A).

**Table 1:** Correct quality identification scores in the whole mouth suprathreshold taste test.

	All		Age 20–39 years				Age 40–59 years				Age ≥ 60 years			
			Male		Female		Male		Female		Male		Female	
Correct quality identification score, median (minimum, maximum)														
S score	10	(8, 10)	10	(9, 10)	10	(9,10)	10	(9, 10)	10	(10, 10)	10	(8, 10)	10	(9, 10)
S1	2	(0, 2)	2	(1, 2)	2	(1, 2)	2	(1, 2)	2	(2, 2)	2	(0, 2)	2	(1, 2)
S2	2	(1, 2)	2	(2, 2)	2	(2, 2)	2	(1, 2)	2	(2, 2)	2	(2, 2)	2	(2, 2)
S3	2	(2, 2)	2	(2, 2)	2	(2, 2)	2	(2, 2)	2	(2, 2)	2	(2, 2)	2	(2, 2)
S4	2	(1, 2)	2	(2, 2)	2	(2, 2)	2	(2, 2)	2	(2, 2)	2	(1, 2)	2	(2, 2)
S5	2	(2, 2)	2	(2, 2)	2	(2, 2)	2	(2, 2)	2	(2, 2)	2	(2, 2)	2	(2, 2)
A score	10	(0, 10)	10	(5, 10)	10	(3,10)	9.5	(2, 10)	10	(0, 10)	9	(1, 10)	8	(0, 10)
A1	2	(0, 2)	2	(0, 2)	2	(1, 2)	2	(0, 2)	2	(0, 2)	2	(0, 2)	2	(0, 2)
A2	2	(0, 2)	2	(1, 2)	2	(1, 2)	2	(0, 2)	2	(0, 2)	2	(0, 2)	2	(0, 2)
A3	2	(0, 2)	2	(1, 2)	2	(1, 2)	2	(0, 2)	2	(0, 2)	2	(0, 2)	2	(0, 2)
A4	2	(0, 2)	2	(1, 2)	2	(0, 2)	2	(0, 2)	2	(0, 2)	2	(0, 2)	2	(0, 2)
A5	2	(0, 2)	2	(1, 2)	2	(0, 2)	2	(1, 2)	2	(0, 2)	2	(0, 2)	2	(0, 2)
C score	10	(4, 10)	10	(6, 10)	10	(4, 10)	10	(4, 10)	10	(4, 10)	10	(4, 10)	10	(4, 10)
C1	2	(0, 2)	2	(0, 2)	2	(0, 2)	2	(0, 2)	2	(0, 2)	2	(0, 2)	2	(0, 2)
C2	2	(0, 2)	2	(0, 2)	2	(1, 2)	2	(0, 2)	2	(0, 2)	2	(0, 2)	2	(0, 2)
C3	2	(0, 2)	2	(1, 2)	2	(1, 2)	2	(1, 2)	2	(0, 2)	2	(0, 2)	2	(0, 2)
C4	2	(0, 2)	2	(1, 2)	2	(0, 2)	2	(1, 2)	2	(1, 2)	2	(1, 2)	2	(1, 2)
C5	2	(1, 2)	2	(2, 2)	2	(2, 2)	2	(1, 2)	2	(2, 2)	2	(1, 2)	2	(1, 2)
N score	10	(4, 10)	10	(6, 10)	10	(7,10)	10	(5, 10)	10	(8, 10)	10	(4, 10)	10.0	(5, 10)
N1	2	(0, 2)	2	(0, 2)	2	(1, 2)	2	(0, 2)	2	(1, 2)	2	(0, 2)	2	(0, 2)
N2	2	(0, 2)	2	(1, 2)	2	(1, 2)	2	(0, 2)	2	(1, 2)	2	(1, 2)	2	(0, 2)
N3	2	(0, 2)	2	(0, 2)	2	(1, 2)	2	(0, 2)	2	(1, 2)	2	(0, 2)	2	(1, 2)
N4	2	(0, 2)	2	(1, 2)	2	(1, 2)	2	(0, 2)	2	(2, 2)	2	(1, 2)	2	(1, 2)
N5	2	(0, 2)	2	(1, 2)	2	(0, 2)	2	(0, 2)	2	(2, 2)	2	(1, 2)	2	(1, 2)
Total score	39	(24, 40)	40	(33, 40)	39	(31, 40)	37	(26, 40)	39	(25, 40)	37.5	(28, 40)	38	(24, 40)
Percentiles														
10	33		35		37		32.1		34		29		36.3	
90	40		40		40		40		40		40		40	

S1-S5 = sucrose solution (molar concentrations: 0.08, 0.16, 0.32, 0.64, 1.28 mol/L); A1-A5 = citric acid solution (molar concentrations: 0.0026, 0.0051, 0.0102, 0.0205, 0.0410 mol/L); C1-C5 = caffeine solution (molar concentrations of: 0.0026, 0.0051, 0.0102, 0.0205, 0.0410 mol/L); N1-N5 = sodium chloride solution (molar concentrations: 0.032, 0.064, 0.128, 0.256, 0.512 mol/L).

**Fig 4:**
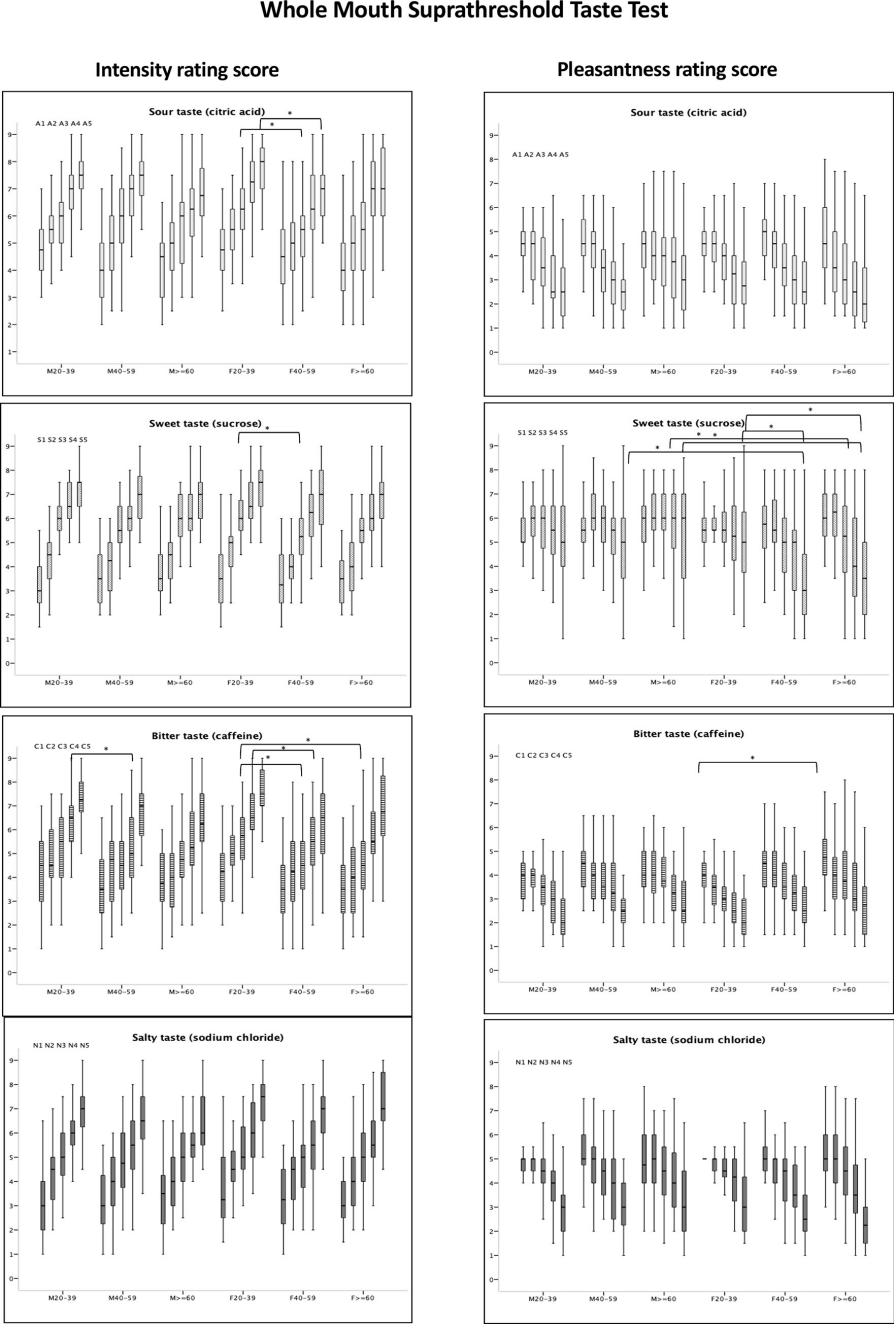
Intensity and pleasantness rating score of whole mouth suprathreshold taste test. Intensity rating scale: 1: not present at all, 2: very slight, 3: slight, 4: definitely present, 5: moderate, 6: moderately strong, 7: strong, 8: very strong, 9: extremely strong. Pleasantness rating scale: 1: dislike extremely, 2: dislike very much, 3: dislike moderately, 4: dislike slightly, 5: neither like nor dislike, 6: like slightly, 7: like moderately, 8: like very much, 9: like extremely. The boxes depict the 25th, 50th, and 75th quartiles. The lines extending parallel from the boxes indicate the range of rating scores. *Significant differences (p<0.05) between two groups.

The intensity and pleasantness rating scores are shown in Fig 4. In general, the intensity rating scores were higher in the 20–39 years age group, regardless of sex. Regarding the pleasantness of tastants, female subjects in the 20–39 years age group found sweet solution more pleasant than older female subjects (S5: vs. 40–59 years, p = 0.004; vs. ≥60, p = 0.02). In contrast, male subjects in the two older age groups found sweetness more pleasant than female subjects in these age groups (S4: p = 0.002 for ≥60 years; S5: p = 0.036 for 40–59 years; p = 0.003 for ≥60 years).

### Taste quad test

The total and individual correct quality identification scores, and intensity rating for the 4 tongue quadrants are shown in Table 2. The total scores for male subjects in the young age group (20–39 years) were significantly higher than those in the two older age groups (20–39 years vs. 40–59 years, 20–39 years vs. ≥60 years, both p<0.001) (Fig 3B). For female subjects, younger subjects also had higher scores than subjects in the 40–59 years age group (p = 0.002). No significant differences in correct quality identification scores and intensity ratings (except bitter tastant) were found between males and females in the same age group. Male subjects ≥60 years had significantly lower correct quality identification scores for the bitter tastant than those of female subjects in this age group (p = 0.033).

**Table 2:** The results of the taste quad test.

	Age 20–39 years				Age 40–59 years				Age ≥60 years			
	Male		Female		Male		Female		Male		Female	
**Correct quality identification score, median (minimum, maximum)**												
**Total score**	80	(40–91)	82	(38–96)	59	(36–86)	63	(36–89)	58	(38–86)	68	(29–90)
**C score**	20	(4–24)	20.5	(10–24)	14.5	(2–23)	14	(2–23)	11	(0–21)	13	(3–23)
quadrant 1	5	(1–6)	6	(1–6)	3	(0–6)	4	(0–6)	3	(0–6)	4	(0–6)
quadrant 2	5	(0–6)	5	(1–6)	4	(0–6)	3.5	(0–6)	3	(0–6)	3	(0–6)
quadrant 3	5	(1–6)	5	(0–6)	4	(1–6)	4	(0–6)	3	(0–6)	4	(0–6)
quadrant 4	5	(1–6)	5	(1–6)	3	(0–6)	3.5	(0–6)	3	(0–6)	3.5	(0–6)
**S score**	22	(9–24)	23	(7–24)	19	(10–24)	19	(11–24)	21	(13–24)	22	(6–24)
quadrant 1	5.5	(1–6)	6	(2–6)	4	(0–6)	4	(1–6)	5	(1–6)	5.5	(0–6)
quadrant 2	6	(1–6)	6	(2–6)	6	(1–6)	6	(2–6)	6	(3–6)	6	(1–6)
quadrant 3	6	(0–6)	6	(0–6)	6	(3–6)	5	(3–6)	6	(3–6)	6	(2–6)
quadrant 4	6	(1–6)	6	(1–6)	4	(1–6)	4	(0–6)	5	(1–6)	6	(0–6)
**F score**	21	(11–24)	22	(8–24)	15	(2–23)	17	(6–24)	16	(5–24)	19	(8–24)
quadrant 1	5	(1–6)	6	(2–6)	4	(0–6)	4	(0–6)	3	(0–6)	5	(0–6)
quadrant 2	5.5	(1–6)	6	(1–6)	4	(1–6)	5	(0–6)	4	(0–6)	5	(1–6)
quadrant 3	6	(1–6)	6	(3–6)	4.5	(0–6)	5	(1–6)	4	(1–6)	5	(2–6)
quadrant 4	5	(1–6)	6	(1–6)	3	(0–6)	4	(0–6)	4	(1–6)	5	(0–6)
**N score**	17	(4–24)	17	(5–24)	12	(3–22)	14	(3–23)	15	(2–23)	15	(3–24)
quadrant 1	4	(1–6)	4.5	(0–6)	3	(1–6)	3	(0–6)	3.5	(0–6)	3	(1–6)
quadrant 2	4	(1–6)	5	(0–6)	3	(0–6)	4	(0–6)	4	(0–6)	4	(0–6)
quadrant 3	4	(0–6)	4	(0–6)	4	(0–6)	3	(0–6)	4	(0–6)	3	(1–6)
quadrant 4	4.5	(0–6)	5	(1–6)	3	(0–6)	3	(1–6)	3.5	(0–6)	4	(1–6)
**Intensity rating score, median (minimum, maximum)**												
**C score**	15.2	(6.2–29.5)	14.6	(5.5–26)	13	(4.8–25.2)	15.1	(6.7–22.8)	13	(6.8–21.5)	15.8	(7.7–28.3)
quadrant 1	3.9	(1.8–8.2)	3.9	(1–7.2)	3.2	(1–7.2)	3.7	(1.2–6.7)	3	(1.2–5.5)	4.1	(1–7.2)
quadrant 2	4.2	(1.8–7.3)	4	(1.5–6.3)	3.4	(1.2–7.7)	3.8	(1–6.3)	3.3	(1.7–5.7)	3.7	(2–7.3)
quadrant 3	3.8	(1.8–6.8)	3.7	(1–6.3)	3.3	(1.5–7.8)	3.5	(1.3–6.2)	3.2	(1.5–5.7)	3.7	(1.7–6.8)
quadrant 4	4	(1.2–7.7)	3.8	(1.3–8.2)	2.8	(1.0–6.2)	3.5	(1.3–6.2)	3.3	(1.5–5.7)	4	(1.7–7.2)
**S score**	15	(6.5–28.3)	15.3	(6.2–25.3)	13.2	(5.3–25.3)	15	(5.8–23.8)	14	(7.3–22.3)	16.1	(6.8–28.7)
quadrant 1	3.6	(1.2–7.3)	3.8	(1.2–8.3)	2.8	(1–6.5)	3.3	(1.3–6)	3.5	(1.7–5.5)	4.1	(1.5–7)
quadrant 2	4.0	(1.8–6.8)	3.8	(1.5–6.8)	3.3	(1.5–7.2)	4	(1.2–6.2)	3.6	(2–6.7)	4.1	(1.8–7.2)
quadrant 3	4	(1.7–7)	3.8	(1.2–6.2)	3.5	(1.7–7.7)	3.8	(1.5–5.8)	3.3	(1.7–5.8)	4.3	(1.3–7.2)
quadrant 4	3.6	(1–7.2)	3.8	(1.7–7.2)	2.9	(1–6.8)	3.1	(1–6.2)	3.3	(1.2–5.8)	4.1	(1–7.3)
**F score**	14	(6.2–29)	16.8	(5.5–29.7)	10.9	(5.2–23.8)	15	(5.8–23.8)	11.5	(5.3–22)	15.3	(7.2–26.5)
quadrant 1	3.4	(1–8)	4.2	(1.3–7.3)	2.4	(1.2–7)	3.2	(1.2–58)	2.8	(1–5.7)	4	(1.3–6.8
quadrant 2	3.6	(1.5–7.3)	3.8	(1.2–7.5)	2.7	(1–6.3)	3.3	(1.3–6.5)	2.8	(1.3–6)	3.5	(1.3–6.5)
quadrant 3	4	(1.5–7.2)	4.4	(1–7.8)	2.7	(1.2–8.2)	3.5	(1.2–8)	2.8	(1.3–6)	3.8	(1.3–7.2)
quadrant 4	3.3	(1–7.5)	3.9	(1.3–7.7)	2.7	(1–6.8)	3.3	(1–6.2)	2.7	(1.2–5.5)	3.6	(1.2–6.8)
**N score**	16.5	(7.3–27.5)	15.8	(6–27.8)	12.6	(5.3–23.3)	16.3	(7–24.5)	13.3	(7.2–24.2)	16.2	(9.2–29.8)
quadrant 1	4.1	(1–7)	4.2	(1.5–8)	2.8	(1–6.7)	3.9	(1.3–6.3)	3.4	(1.2–6)	4.3	(1.3–7.7)
quadrant 2	4.2	(2.0–6.7)	3.9	(1.3–7.7)	3.3	(1.2–7.5)	3.8	(1.5–6)	3.3	(1.5–6.8)	4.3	(1.7–7.2)
quadrant 3	4.4	(2.2–6.7)	4.3	(1.7–72)	3.3	(1.5–7.8)	4	(1.3–6.3)	3.3	(1.8–6.5)	3.8	(2.2–7.5)
quadrant 4	4	(1–7.3)	4.2	(1.3–7.8)	2.8	(1–6)	3.8	(1.3–6.2)	2.8	(1.5–6.3)	4.4	(1.3–7.5)

**C**: 0.015 mol/L citric acid solution (sour); **S**: 0.49 mol/L sucrose solution (sweet); **F**: 0.04 mol/L caffeine solution (bitter); **N:** 0.31 mol/L sodium chloride solution (salty).

**Fig 5:**
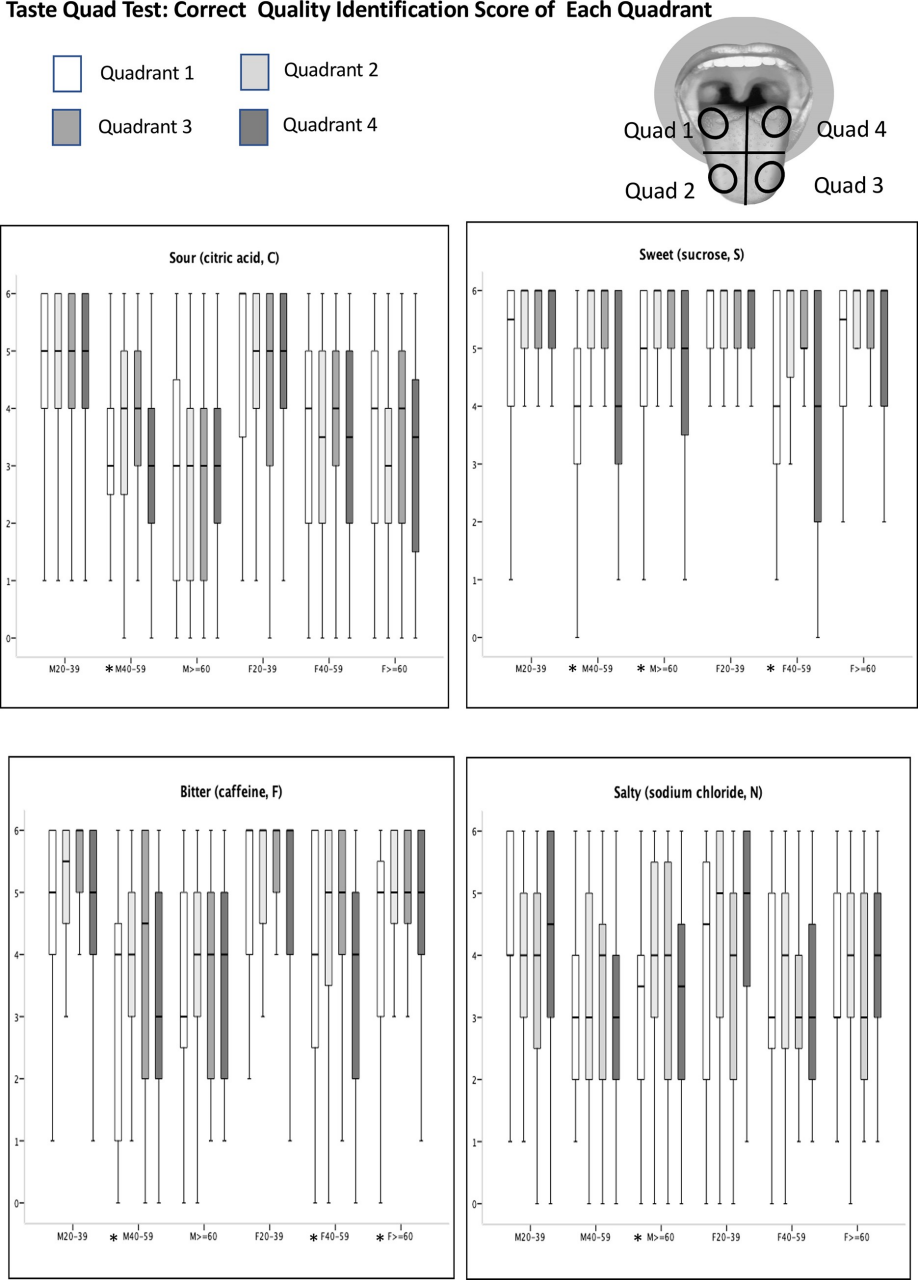
Plots of the correct quality identification scores for four quadrants in the taste quad test. The boxes depict the 25th, 50th, and 75th quartiles. The lines extending parallel from the boxes are used to indicate the range of score. * significant differences (P<0.05) in correct quality identification score among 4 quadrants.

Regarding individual quadrants, for the anterior tongue, age was found to significantly influence identification of sour and bitter tastants (quadrants 2 and 3), especially in male subjects (Fig 5). When the correct quality identification scores were compared among the 4 tongue quadrants, we found no differences among quadrants in the 20–39 years age group. For the anterior tongue (quadrants 2 and 3) of subjects aged 40–59 years, the correct quality identification scores were significantly higher compared with those of the posterior tongue (quadrants 1 and 4) for sour, sweet, and bitter tastant solutions in males (p = 0.025, <0.001, = 0.02, respectively), and for sweet and bitter tastants in females (both p <0.001). Furthermore, males aged over 60 years had better identification scores of sweet and salty tastants for the anterior tongue compared with the posterior tongue (p<0.001 and = 0.012, respectively). Females aged over 60 years had better identification scores of bitter tastant for the anterior tongue compared with the posterior tongue (p = 0.019).

**Fig 6:**
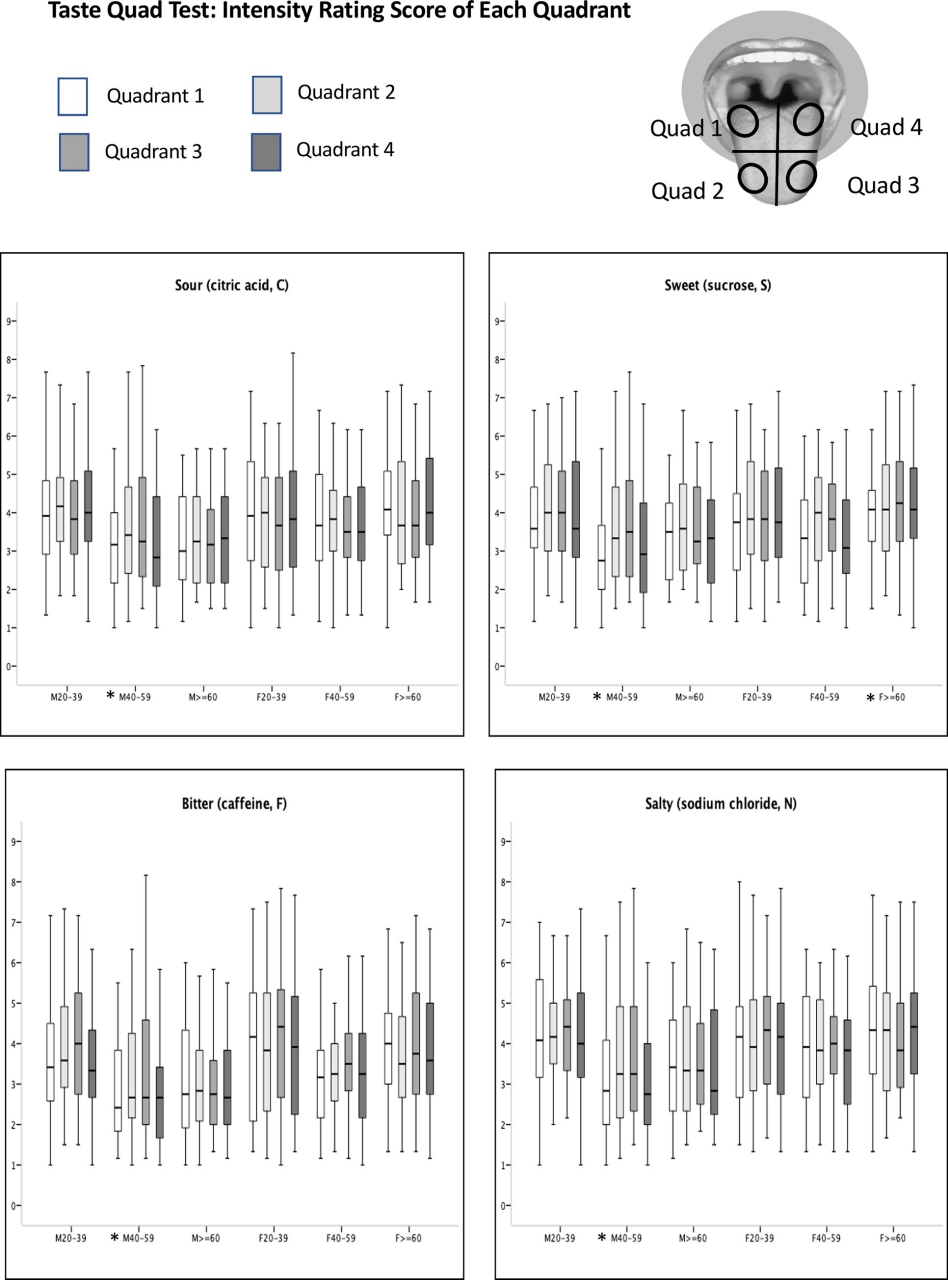
Plots of the intensity rating scores for four quadrants in the taste quad test. Intensity rating scale: 1: not present at all, 2: very slight, 3: slight, 4: definitely present, 5: moderate, 6: moderately strong, 7: strong, 8: very strong, 9: extremely strong. The boxes depict the 25th, 50th, and 75th quartiles. The lines extending parallel from the boxes indicate the range of scores. * Significant difference (P<0.05) in intensity rating score among 4 quadrants.

The intensity rating scores of the anterior tongue (quadrants 2 and 3) were significantly higher than those of the posterior tongue (quadrants 1 and 4) for all 4 tastants in males aged 40–59 years (sour, sweet, bitter, salty: p<0.001, <0.001, = 0.016, = 0.019, respectively). In addition, the anterior tongue of females aged over 60 years had higher intensity rating scores compared with those of the posterior tongue for sweet tastant (p<0.001) (Fig 6).

## Discussion

It has been shown that aging results in a decline in taste function and that this age-related decline is probably tastant-dependent [4]. In the present study, we found a similar decline in healthy Taiwanese subjects in terms of the total correct quality identification scores of the whole mouth suprathreshold taste test. However, our findings differed from those reported by Yang et al. [8]. They did not find any age-related decline in the scores of the three-drop test in healthy Chinese subjects. In a study by Doty et al. [16], who employed the same procedures as those in this study, the scores of the whole mouth test were not significantly different between males and females. Similarly, our study also showed no sex differences in each of the age groups.

Regarding taste qualities, the ability to identify sweet taste was reported to persist well into old age [13]. Our whole mouth test results also showed that aging did not affect the ability to identify saltiness. Moreover, we did not find any sex differences with respect to individual taste qualities, except that women had better identification scores for sweetness than men in the 40–59 years age group. In contrast, Welge-Lussen et al. [13] reported that women were slightly better than men at identifying different tastes in the taste strip test. In a study conducted by Pingel et al., the authors recommended using the 10th percentile of taste scores as a normative value [14]. The 10th percentiles of correct quality identification scores of the whole mouth suprathreshold taste test in the present study tended to decline with age (scores in males aged 20–39, 40–59, ≥60 years were 35, 32.1, and 29, respectively; scores in females aged 20–39, 40–59, ≥60 years were 37, 34, and 36.3, respectively).

With respect to the intensity and pleasantness ratings, it was reported that young subjects rated bitter and sour tastes more intensely than older subjects, but not sweet and salty tastes [7]. Consistent with this finding, we found similar results for the whole mouth test, particularly for the bitter taste. However, in our study, subjects aged 20–39 years also rated sweet taste more intensely than older subjects. While females were found to rate bitter and sour tastes more intensely than males [7], our results showed that all individual tastes were rated similarly intense regardless of sex. However, female subjects in the 20–39 years age group found sweet solution more pleasant than females in the older age groups. In addition, male subjects found sweet solution more pleasant than female subjects in the same age group. Winpenny et al. [17] conducted a systemic review and reported a decrease in sugar consumption through adolescence to adulthood. Graff et al. [18] investigated the perceived sweetness intensity and pleasantness of sucrose in children, adolescents, and adults. They found that adolescents showed a lower sensitivity to sucrose than adults, and adolescents had a higher optimal preferred sucrose concentration than adults. These findings might explain the decreased preference for sweet taste with age.

The regional taste test has been used to detect gustatory blind regions on the tongue surface [14]. A few studies reported age-related decline in taste ability in localized regions of the tongue. Pingel et al. [14] found that regional taste test scores declined with age for both the right tongue and the left tongue. They also found that women were more sensitive to tastant solutions than men on both sides of the tongue, but the left tongue was more sensitive to tastant solutions than the right tongue in elderly subjects. Interestingly, Doty et al. [19] reported a slight age-related decline in taste function at the front surface of the tongue during middle age, which became more pronounced with age but without sex differences. In another study on the effect of age, sex, and tongue side on electrogustometry thresholds, Pavlidis at al. [20] found that the taste acuity of the tongue decreased with age, possibly because of a decrease in the density of fungiform papillae. In our study, correct quality identification scores were not significantly different across the 4 quadrants of the tongue for male and female subjects aged 20–39 years. Nevertheless, we found an age-related decline in taste function on the posterior tongue surface, especially for sweet and bitter tastes. In addition, women had higher correct quality identification scores for bitter taste compared with men, particularly among older subjects (≥60 years).

Taste function has rarely been investigated in Asian populations. It is widely believed that differences in eating habits, diet, and culture among populations affect the results of taste tests [8, 21, 22]. Yong et al. [8] proposed that Asians are more taste-sensitive. Shu-Fen et al. [22] reported that Indians had higher recognition thresholds for all taste qualities compared with Chinese subjects. Our results were obtained from a Taiwanese population, which has considerable differences in eating habits, diet, and culture compared with Western populations, Indians, and other Chinese populations. Future cross-cultural studies are needed to clarify these population-related differences.

In conclusion, our results showed that both age and sex affected taste function in healthy Taiwanese. The total correct quality identification scores of the whole mouth suprathreshold taste test and the quad taste test decreased with age, but the influences were not uniform. Moreover, the ability to identify sweet and salty tastes was not affected by age. Our younger subjects tended to rate tastant solutions more intensely than the older subjects. In addition, the age-related decline in taste function occurred mostly on the posterior tongue surface. Our results were somewhat in agreement with the findings of previous studies, but also demonstrated novel findings. Therefore, in the measurement of taste function and interpretation of results, multiple factors such as age, sex, tongue region, taste quality, and ethnicity should be taken into consideration.

## Supporting information

S1 Data (XLSX)

S2 Data (XLSX)

S3 Data (XLSX)
